# Performance of the Cement Grouting Material and Optimization of the Mix Proportion for the Free Section of the Prestressed Anchor Bar

**DOI:** 10.3390/ma16206819

**Published:** 2023-10-23

**Authors:** Chaoguang Wu, Zhiya Chen, Xuemin Zhang, Zikun Li, Lichuan Wang, Bin Ouyang, Jin Liu

**Affiliations:** 1 School of Civil Engineering, Central South University, Changsha 410075, China; chaoguangwu@csu.edu.cn (C.W.); chenzy@csu.edu.cn (Z.C.); zhangxm@csu.edu.cn (X.Z.); wlc773747@126.com (L.W.); 214802079@csu.edu.cn (J.L.); 2 School of Transportation, Central South University, Changsha 410075, China; 3 Key Laboratory of Heavy-Haul Railway Engineering Structure, Ministry of Education, Central South University, Changsha 410075, China; 4 Guizhou Bridge Construction Group Co., Ltd., Guiyang 550000, China; gqzgb@gzql.cn

**Keywords:** prestressing anchors, grouting material, water-reducing agent, flowability, multi-objective optimization

## Abstract

Increasing the water–cement ratio and water-reducer dosage of cement slurry enhances its fluidity. However, a high water–cement ratio diminishes the beneficial effects of water reducers on fluidity. The stone content of the slurry decreases as the water-reducer dosage increases. Additionally, the water–cement ratio significantly affects stone content. However, when the water–cement ratio exceeds a threshold value, stone content decreases. Furthermore, the threshold value of the water–cement ratio decreases with increasing water-reducer dosage. Without the addition of the water reducer, as the water–cement ratio increases the overall integrity of the grout stone decreases. The addition of the water reducer alters the surface pore distribution, wherein “uniform small pores” change to “localized large pores.” Based on the multi-objective optimization of Matlab, the recommended optimal mix composition for a slow-setting cement slurry is a water–cement ratio of 0.25 and water-reducer dosage of 1.5%. With the use of this optimized mix composition, the stone content and compressive strength increase by 7.8% and 145.6%, respectively, compared to those obtained using the recommended mix ratio in the specifications. Additionally, all relevant performance parameters meet the requirements specified by previous standards.

## 1. Introduction

**Figure 1:**
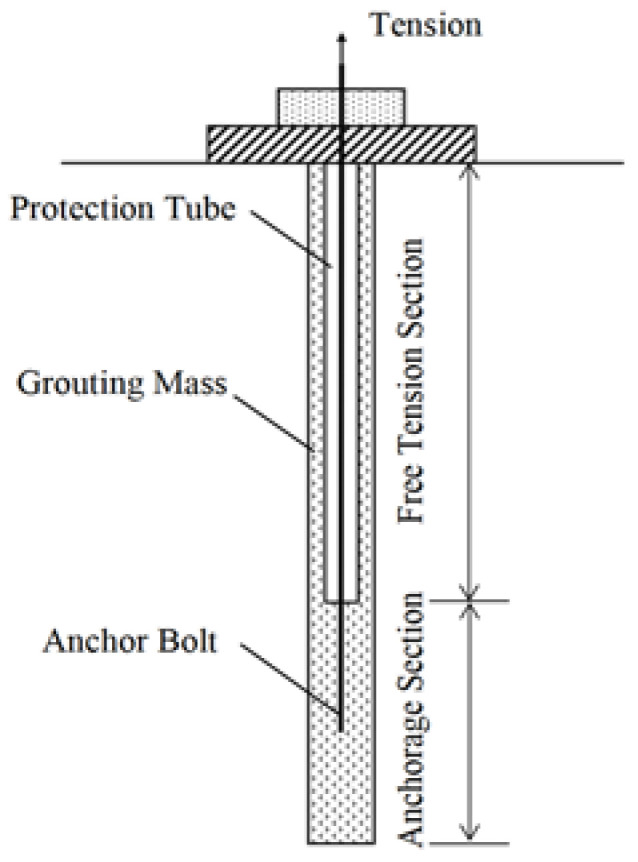
Prestressed anchor.

Using an anchor rod support is one of the most commonly employed techniques in the field of geotechnical support [1,2]. In tunnel construction, under the conditions of a significant burial depth, high stress, soft rock, and fractured surrounding rocks, [3,4] the requirements of the anchoring force and load-bearing capacity of rock bolts are significant for the stability control of the surrounding rocks in the tunnel. Currently, prestressed anchor bolts are widely used as critical active support systems in tunnel construction involving high stresses and fractured surrounding rocks. In practical projects, it is a common practice to first complete the grouting of the free section of the anchor hole before tensioning the anchor bolts, as illustrated in Figure 1. Therefore, slow-setting grouting materials should be used for the free section. However, the inadequate performance of the slow-setting cement slurry can adversely affect the tension of the anchor bolts and may result in slurry rupture and subsequent failure [5] of the bolt anchorage, as indicated in previous studies. In China, current standards [6,7] clearly define the key techniques for cement-based grouting materials used for the grouting of the free sections of the prestressing anchor bolts. In general, the recommended value for the water–cement ratio is set at 0.5. However, the standards do not provide specifications for engineering indicators such as the fluidity and stone content, which affect the performance of the slow-setting cement-based materials. As a result, despite using grout that meets the standard requirements, an inadequate fluidity or stone content may be employed in certain projects, leading to the incomplete filling of the anchor holes and affecting the tension quality of the prestressing anchor bolt. Therefore, it is necessary to systematically develop a slow-setting cement-based grouting material for the free section of the prestressing anchor bolt used in tunnels that exhibits a good working performance and to determine its optimal mixture proportion.

Slow setting cement-based grouting materials can effectively delay the hydration reaction of cement, prolong the initial setting time, and have no adverse effects on the mechanical properties of the grouting materials in the later stage, which is conducive to maintaining the plasticity and long-term strength of the grouting materials and has good grouting properties. At present, there are many types of cement retardants commonly used in domestic and foreign projects. Inorganic retardants mainly include boric acid, borax, and zinc chloride, etc., and organic retardants mainly include lignosulfonate, carbohydrate and carbohydrate, polyol and hydroxy-carboxylic acid (salt), etc. [8,9]. Most scholars in the industry [10,11,12,13,14] believe that there is an optimal dosage of retarder, and its type also has a great impact on the quantitative and mechanical indexes of grouting. Relevant researches have also obtained some important conclusions on the effects of inorganic and organic retarders on the properties of grouting materials. You et al. [15,16,17,18,19,20] focused on the influence of retarding agents such as borax, boric acid and polyphosphate on the hydration reaction temperature of cement, and concluded that the three retarding agents have different degrees of influence on the heat release of retarding hydration, with boric acid having the best retarding effect and polyphosphate having the worst. Vasumithran et al. revealed that mixtures containing silica fume exhibited poor workability only at low water–cement ratios but demonstrated improved strength compared to other types of mixtures [21]. Compared to cement grout containing only silica fume, grout containing fine sand and fly ash exhibit a lower early strength. Lin et al. investigated the influence of re-dispersible latex powder and hydroxypropyl methyl cellulose on the physical and mechanical properties of high water-to-cement-ratio cement–sodium silicate grouts [22]. They evaluated and compared the rheological characteristics, setting time, compressive strength, resistance to sulfate attack, and permeability of the grouts. The results indicated that with the addition of these two polymers, the water glass–cement grouts exhibited a favorable performance and durability. Sonebi developed statistical models for various ranges of the water–cement ratio, high-range water reducer dosage, silica fume replacement ratio, and viscosity-modifying admixture dosage [23]. 

In summary, existing studies on the improvement of grouting materials in the free section of tunnel prestressed anchor rod mainly include mixing single retarder or compound retarder, but some of the single retarder and compound retarder have negative effects on the performance of grouting materials, and polycarboxylate superplasticizer is an additive with excellent dispersion and compatibility. It can maintain the high flow shaping of the slurry at low dosage, maintain excellent engineering performance at low water-binder ratio, and have good compatibility with cement, so it should be used as the research direction of optimizing the grouting material of tunnel anchor with slow setting. However, polycarboxylic acid water reducer is rarely involved in the existing research. Therefore, this study employs laboratory experiments, scanning electron microscopy (SEM) studies, and Matlab multi-objective optimization methods to analyze the properties and optimal mix proportions of slow-setting cement–polycarboxylate water-reducing agent grouting materials. This study aims to provide guidance for the widespread application of cement–polycarboxylate water-reducing agent grouting materials in tunnel prestressed anchor rods.

### 1.1. Indicators for Improving the Slow-Setting Cement-Based Grouting Materials

#### 1.1.1. Flowability

**Figure 2:**
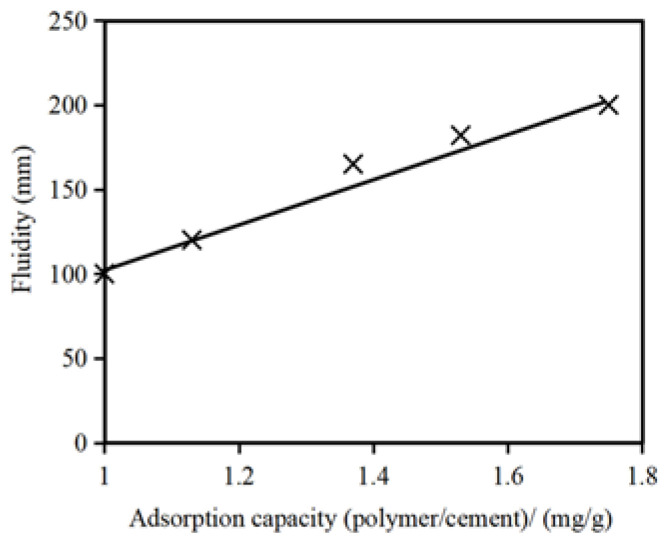
Relationship between the polymer adsorption capacity and fluidity of the slurry.

Flowability refers to the macroscopic manifestation of the molecular diffusion of grouting materials during the grouting process. It reflects the pumpability and stability of the reactive grout slurry and serves as a measurable indicator of workability. The current general consensus in the academic community regarding the enhanced flowability of cementitious slurries by polycarboxylate water-reducing agents is as follows. Polycarboxylate-based water-reducing agents have longer side chains, rendering them less susceptible to coverage by cement hydration products. As a result, they can adsorb onto cement particles or cement hydration products, creating steric hindrance effects, which allow for a prolonged dispersion and maintenance of the dispersion of the cement particles, leading to flowability retention. Therefore, the adsorption amount of the polymers in the water-reducing agents is critical for both the water-reducing performance and flowability enhancement in cementitious slurries. Figure 2 presents a curve illustrating the relationship between the flowability of cement slurry and the adsorption amount of the water-reducing agent polymer. A pronounced positive correlation exists between the flowability of the slurry and the adsorption amount of the water-reducing agent polymer; the flowability of the cement slurry is dependent on the adsorption capacity and dosage of the polymer.

To measure the flowability of the cement slurry, a prepared single-liquid slurry is poured into a truncated cone mold, which is then lifted. After the specimen slump stabilizes, the maximum diameter in the two perpendicular directions of the flowing portion is measured using a steel ruler. The average value is considered as the flowability of the neat cement slurry. At different mix ratios, the flowability of the single-liquid cement slurry can be categorized into the following three states: plastic, plastic-flow, and fluid states. For the different flow states, the flowability measurements, state descriptions, and images of their respective conditions are presented in Table 1. Based on the three aforementioned flow states, the minimum required flowability for cement slurry is ≥120 mm, indicating that the cement slurry must be in a fluid state.

#### 1.1.2. Stone-Formation Rate

Cement-based grouting materials are inevitably subjected to a certain degree of consolidation and shrinkage during the setting and hardening processes. The magnitude of the shrinkage directly determines the filling rate of the grouting holes, and the extent of this shrinkage can be characterized by the stone content rate. The 24-h stone-formation rate R_s_ was calculated according to Equation (1).

(1) $$R_{s}=\frac{V_{s}}{V}\times 100\%$$

where *R_s_* is the rate of stone formation (%); *V_s_* is the volume of the stone body at 24 h (cm^3^); and *V* is the volume of the dumped slurry (cm^3^).

## 2. Materials and Methods

### 2.1. Test Materials

#### 2.1.1. Cement

Ordinary Portland cement (P.O42.5) was used in the experiments; its physical properties are listed in Table 2. In the existing studies [24,25], the content of water reducing agent is all within 2%, and excessive inclusion of water reducing agent in cement slurry will affect the durability of slurry.

#### 2.1.2. Water-Reducing Agent

The experiments utilized a polycarboxylic acid water-reducing agent with a recommended dosage of 2.0% based on the mass of cement. The solid content of this water-reducing agent was 13.37%; the pH was 6.7, and the water-reduction rate was 19.80%.

### 2.2. Experimental Design

This study employed a controlled variable method to investigate the combined effects of two factors, namely, the water–cement ratio and water-reducing agent dosage, on the flowability, stone content, 28-day compressive strength, initial setting time, and final setting time of the cement slurry. A total of 15 cement slurry mixtures were designed, as summarized in Table 3. The maximum recorded duration for the initial and final setting times was 1440 min. If a slurry did not set within 1440 min, it was considered ineffective.

## 3. Results

### 3.1. Analysis of the Fluidity of the Slurry

**Figure 3:**
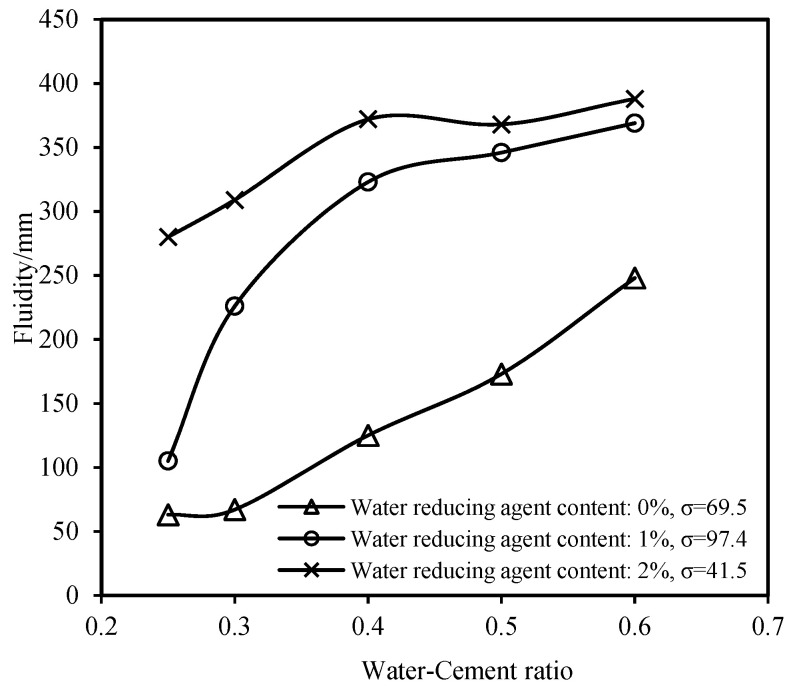
Fluidity curves with respect to the water–cement ratio.

Figure 3 presents the variation in the fluidity of the slurry with different water–cement ratios for various dosages of the water-reducing agent. The incorporation of the water-reducing agent into the cement enhances the fluidity of the cement slurry. Additionally, the sensitivity of the fluidity of the slurry to the dosage of the water-reducing agent is significantly influenced by the water–cement ratio. When the water–cement ratio (w) is ≤0.3, a 1.0% dosage of the water-reducing agent results in a relatively small enhancement of the fluidity of the slurry; in this case, it is advisable to appropriately increase the dosage of the water-reducing agent to ensure a sufficient fluidity of the slurry. However, when the water–cement ratio (w) is >0.3, the different dosages have a similar effect on the enhancement of the fluidity of the slurry. In this case, it is recommended to moderately reduce the dosage of the water-reducing agent while ensuring that the fluidity requirements of the slurry are met to allow for the economic considerations.

### 3.2. Analysis of the Slurry Sedimentation Rate

**Figure 4:**
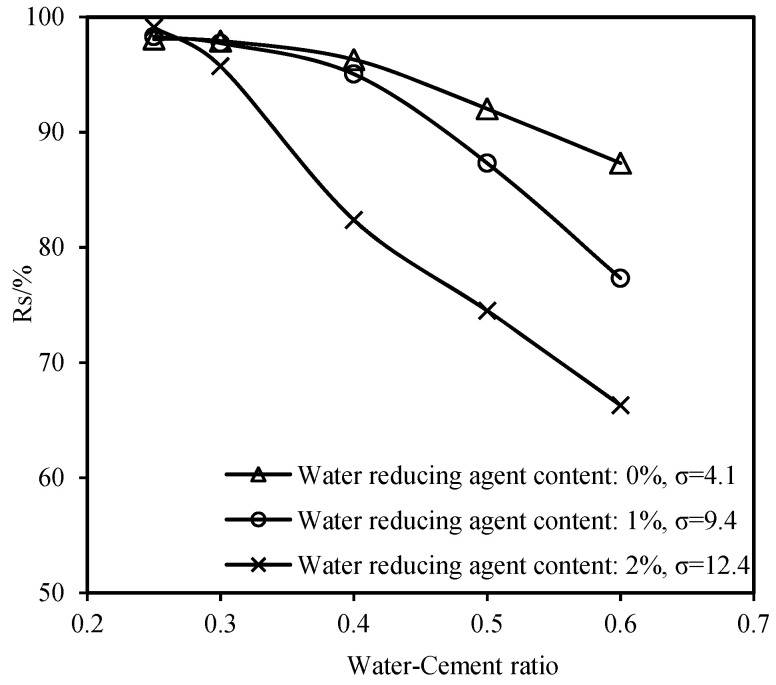
Stone rate with respect to the water–cement ratio.

Figure 4 depicts the relationship between the 24-h stone content and the water–cement ratio for different dosages of the water-reducing agent. The variation in the stone content of the slurry is relatively insignificant when the water–cement ratio (w) is between 0.25 and 0.3. However, when the water–cement ratio (w) is increased to >0.3, a significant reduction in the stone content of the cement slurry is noted. Additionally, the increase in the dosage of the water-reducing agent results in a decrease in the stone content of the slurry. Notably, a critical value of the water–cement ratio that affects the stone content of the cement slurry exists; when the water–cement ratio is below this critical value, the stone content of the cement slurry remains relatively constant. However, when the water–cement ratio exceeds this critical value, the stone content decreases significantly with further increases in the water–cement ratio. In this study, this critical value is referred to as the threshold of the water–cement ratio. The experimental results indicate that the dosage of the water-reducing agent affects the magnitude of the threshold of the water–cement ratio. Table 4 presents the maximum values of the threshold of the water–cement ratio and stone content rate for various dosages of the water-reducing agent. The threshold of the water–cement ratio decreases as the dosage of the water-reducing agent increases, whereas the upper limit of the stone content rate increases.

### 3.3. Analysis of Other Workability Performance Indicators of the Slurry

**Figure 5:**
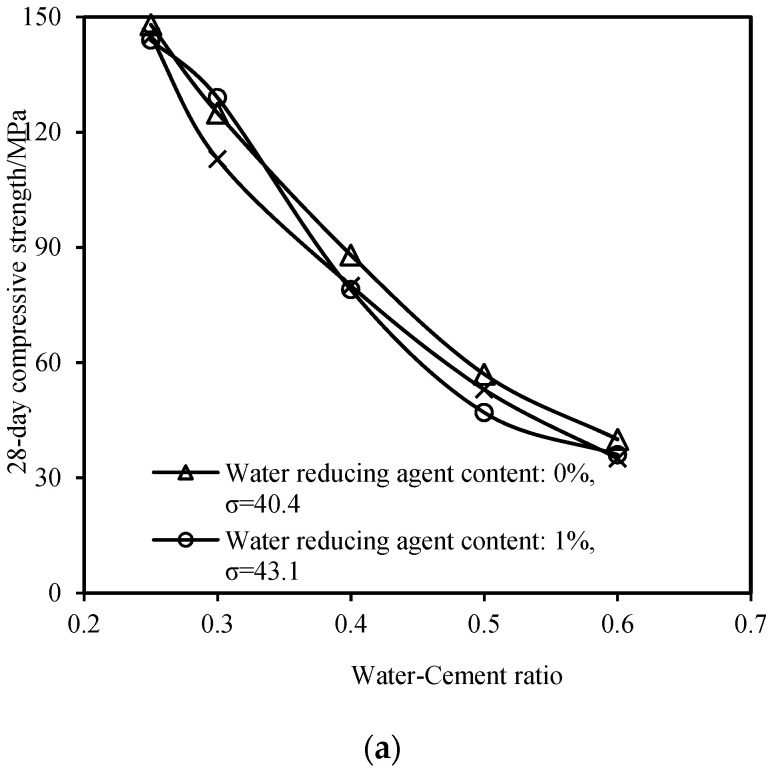

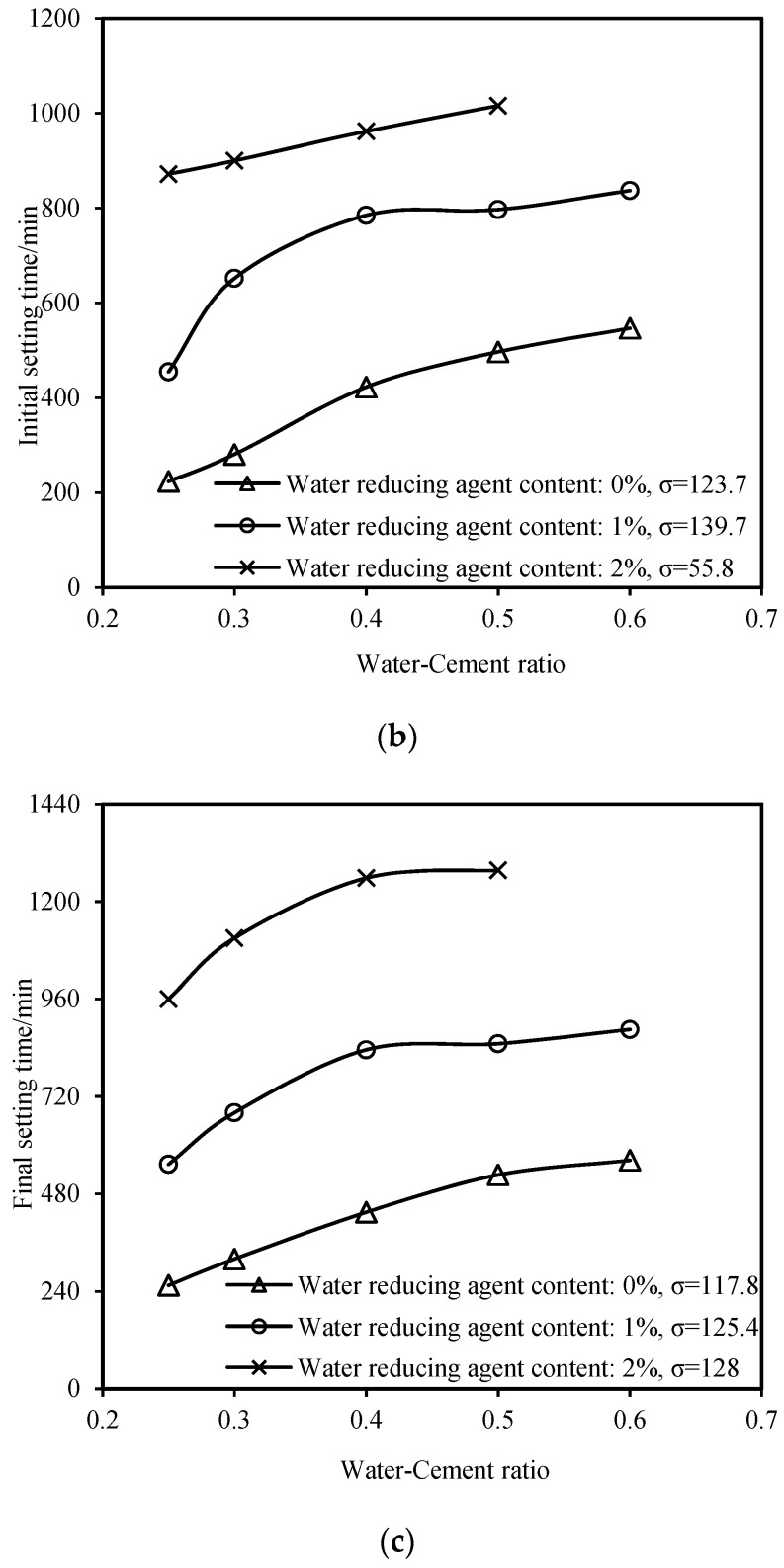
Variations in the other performance indices with respect to the water–cement ratio: (**a**) 28-day compressive strength, (**b**) initial setting time, and (**c**) final setting time.

The workability performance indicators of the slow-setting cement-based grouting material also include the 28-day compressive strength, initial setting time, and final setting time. Figure 5a presents the variation in the 28-day compressive strength of the grout with different dosages of the water-reducing agent and water–cement ratios. The 28-day compressive strength of the grout decreases as the water–cement ratio increases. The addition of the water-reducing agent has a minimal impact on the 28-day compressive strength of the grout. Figure 5b,c depict the variation curves of the initial and final setting times of the slurry at different dosages of the water-reducing agent and water–cement ratios, respectively. An increase in both the water–cement ratio and water-reducing agent dosage leads to an increase in the initial and final setting times of the slurry. Moreover, when the water–cement ratio (w) is 0.6 and the dosage of the water-reducing agent is 2%, the initial setting time of the slurry exceeds 1440 min, indicating that at this composition, the slurry cannot set and solidify.

### 3.4. SEM Analysis of the Slurry Stone Formation

**Figure 6:**
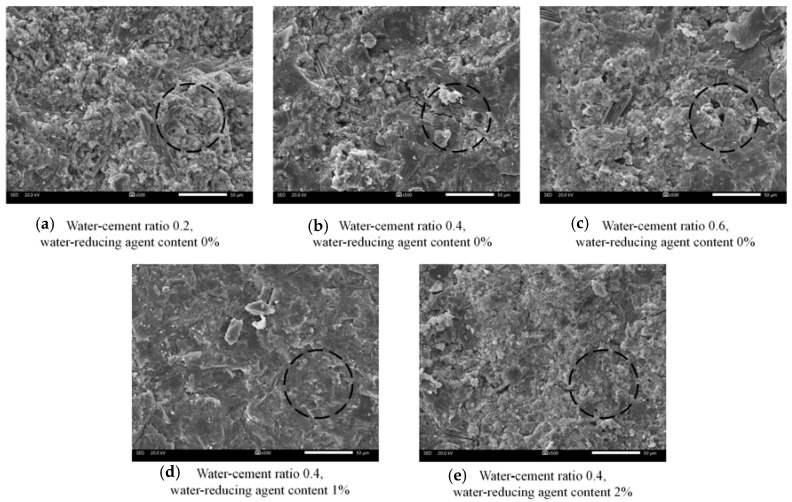
SEM image of a serous stone body (The black circles reflect the local porosity of the stone body).

Figure 6 presents the SEM micrographs of the slurry stone formation under various critical experimental conditions, at a magnification of 500×. Figure 6a–c demonstrate that without the addition of a water-reducing agent, as the water–cement ratio increases, the overall integrity of the slurry stone formation decreases; the porosity on the surface of the stone formation increases (Circled part in the figure), and a high porosity is less conducive to the bonding between the grouting material and the anchor rod as well as the surrounding rock. Figure 6b,d,e demonstrate that when the water–cement ratio remains constant, the addition of the water-reducing agent causes a transition in the pore distribution pattern on the surface of the slurry stone; “evenly distributed small pores” transform to “localized large pores”. The overall integrity of the stone formed outside the pore-distribution region is improved.

## 4. Discussion

The “Technical Specification for Anchor Bolt Support in Railway Tunnels” (Q/CR 9248-2020) in China specifies the main technical indicators for slow-setting cement-based grouting as follows: initial setting time of >8 h, final setting time not exceeding 24 h, and a 28-day compressive strength of >35 MPa. In current engineering practices, to ensure the fluidity of the grout, the commonly used water-to-cement ratio for cementitious grouts in anchoring projects ranges from 0.5 to 1.0. However, grouts with high water-to-cement ratios often exhibit a low stone content, prolonged setting times, and an insufficient strength of the stone bodies. This frequently leads to issues such as the incomplete filling of anchor holes or grouting reinforcement holes, excessive displacement of the surrounding rock, and significant defects resulting in anchor bolt failures. Therefore, in this section, by evaluating the performance indicators of slow-setting cementitious grouts as well as employing multiple regression analysis and multi-objective optimization methods, an optimal mix proportion for slow-setting cementitious grouts that meets both the specifications and practical engineering requirements for all performance indicators is obtained.

### 4.1. Minimum Requirements for the Flowability and Stone-Formation Rate of Grouting Materials

As listed in Table 1, when the flowability of cement slurry is ≥120 mm, the slurry can maintain a fluid state. However, to ensure satisfactory grouting effects in practical engineering applications, an adequate degree of the flowability margin should be ensured. Considering that the current specification does not make provisions for the degree of flow of the cement net slurry, actual field experiment results are incorporated in this study, indicating that when the degree of flow of the cement net slurry is ≥200 mm, it can meet the requirements of a project without plugging the pipe or the grouting is not full.

Considering that the current standards in China have not explicitly defined the requirements for the stone content rate of cement slurry, experiments regarding the stone-formation rate as an important indicator to measure the degree of shrinkage during the solidification process of cement slurry were conducted in this study on the cement slurry mix ratios recommended in the relevant specifications [6]. The stone-formation rate of the cement slurry with a water–cement ratio of 0.5 was 92%. Considering a practical engineering experiment, achieving a stone-formation rate of more than 97% in the cement slurry can result in a better filling rate in the anchor holes.

### 4.2. Verification of the Optimal Proportion of the Cement Slurry Mix Based on Multi-Objective Optimization

#### 4.2.1. Multivariate Regression Analysis of the Slurry Performance

Based on the experimental results presented in the third section, it is challenging to identify a slurry mixture that satisfies all of the performance criteria from the 15 tested combinations. To determine the optimal mixture for the cement-based grouting material with slow-setting characteristics, a conventional quadratic regression model is employed. Utilizing statistical package for the social sciences in conjunction with a stepwise regression approach, a multivariate regression analysis is conducted on the data related to various performance parameters of the slurry discussed in the previous section. This analysis aims to derive the optimal fitting equations for both slurry flowability and stone-formation rate. The main factors influencing the construction performance of slow-setting cement-based grouting materials and the degree of change include: (1) water–cement ratio (*X*_1_ = W/C), where C is the cement content, and W is the amount of water used for mixing. The value adopted in engineering applications is 0.225–0.7; (2) polycarboxylic acid water-reducing agent dosage, *X*_2_ = 0–2.0%. The fitting equations and their corresponding fitting performance are presented in Table 5. According to the fitting results, the significance level values of all variables, P, are less than 0.001, indicating that the regression is extremely significant; the adjusted R^2^ values of all variables are above 0.8, and as per general conventions, the closer the R^2^ value to 1, the better the fit. In conclusion, the multiple regression equations presented in Table 5 demonstrate a favorable fitting performance.

During the construction process of the tunnel anchor bolt, to achieve a good short-term fluidity during grouting and an early strength within a specified time after grouting for cementitious slurries with delayed-setting characteristics, it is necessary to optimize the mixture ratio of the cementitious slurry. For practical engineering applications, the two core performance indicators, namely, strength and setting time, should be considered in the optimization process of the mixture ratio. This involves solving a multi-objective optimization problem where both indicators should be optimized simultaneously. For this purpose, a mathematical approach known as multi-objective programming should be employed to obtain the optimized mixture ratio for the cementitious slurry for achieving an improved performance.

Considering that the optimization problem in this study involves linear objective functions and constraints, the “fgoalattain” function from the MATLAB optimization toolbox is a suitable choice for solving this problem. This can be used to achieve the optimization goals by attaining specific target levels for multiple objectives while satisfying the given constraints. To ensure the stability of the cementitious grout materials in anchor holes and a timely active support during tunnel excavation, this study considers the grout strength and final setting time as the objective functions. The target of the grout strength is set at 100 MPa, and the final setting time is set at 900 min. Other performance requirements are treated as constraints. This study aims to find the optimal condition for the performance of the grout material that satisfies these objectives and constraints. This optimization process aims to balance the desired high strength of the grout with an acceptable setting time while meeting all of the other performance requirements. The optimal performance objective function and constraints are expressed as follows:

(1) Objective functions

- Minf_28-d compressive strength_ = 220.367 − 315.203*X*_1_ − 320*X*_2_
- Minf_Final setting time_ = −10.167 + 1005.366*X*_1_ + 39,450*X*_2_

(2) Constraint conditions

- Fluidity: f_Fluidity_ ≥ 200 mm
- Stone-formation rate: f_Stone rate_ ≥ 97%
- Initial setting time: f_Initial setting time_ ≥ 480 min

Using the Matlab toolbox, with the given objective function and constraints, a program was developed to obtain the following optimal composition of the slurry: water–cement ratio of *X*_1_ = 0.25 and admixture dosage of *X*_2_ = 1.5%.

#### 4.2.2. Experimental Verification of the Optimal Slurry Mix Ratio

After obtaining the optimal slurry mix ratio through multi-objective optimization, the predicted performance of the slurry was verified through laboratory experiments. Table 6 presents a comparison between the predicted performance based on the optimal mix ratio and the actual experimental performance of the slurry.

Table 6 indicates that the experimental results obtained with the optimal slurry composition meet the requirements of the on-site construction performance indicators. The regression analysis for the various slurry performances demonstrates good fitting results, with error rates of less than 5% for most of the performance indicators compared to the actual test values. The fluidity and initial setting time had a relatively large error. To reduce the error, the optimal performance test was repeated three times with the same ratio, and the average value was considered as the final value.

### 4.3. Comparison between the Proposed and Standard Recommended Ratios

#### 4.3.1. Slurry Performance Comparison

**Figure 7:**
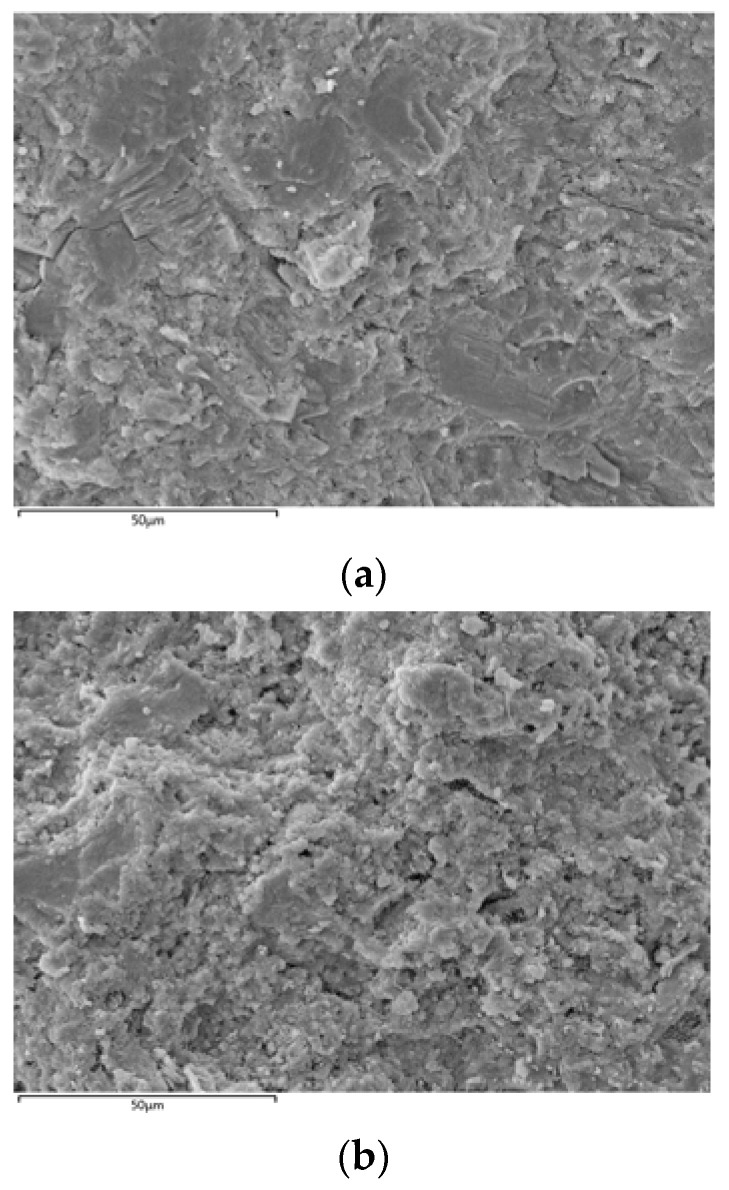
SEM image of the serous stone body using values (**a**) proposed in this study, and (**b**) the standard recommended.

After obtaining the optimal mix ratio for the slow-setting cementitious grout, a comparative test was conducted in this study using the recommended mix ratio as per the relevant standards [6]. The results of the tests are presented in Table 7. The comparative test results indicate that compared to those of the cementitious grout with -the mix ratio recommended by the standards, the characteristics of the cementitious grout with the mix ratio proposed in this study improved significantly. Specifically, the use of the recommended mix ratio led to a 50.9% increase in the flowability, a 7.8% increase in the stone-formation rate, and a significant increase of 145.6% in the compressive strength. All of these values meet the engineering requirements. However, note that the setting time of the proposed mix is longer than that of the standard recommendation; however, both initial and final setting times are within the permissible range defined by the standards. Figure 7 presents the SEM images of the stone formations in the two types of the cementitious grouts, which indicate that the stone formations in the cementitious grout with the mix ratio proposed in this study exhibit smaller pores and a higher overall integrity compared to the stone formations in the cementitious grout with the mix ratio recommended by the standards.

#### 4.3.2. Slurry Durability Comparison

In addition, durability is also an important index to evaluate the quality of the slurry, and chloride ion content is the main index to measure the durability of the slurry. The “Technical Specification for Anchor Bolt Support in Railway Tunnels” (Q/CR 9248-2020) in China requires that the chloride ion content in cement-based slow-setting grout is less than 0.06%. Therefore, this paper monitors the chloride ion content of cement grout with the recommended mix ratio proposed in this paper and the recommended mix ratio in the specification according to this standard, and the results are shown in Table 7. According to the data, after the addition of water reducing agent, the chloride ion content of the cement slurry rose to 0.025%, but it was still within the allowable range of the specification.

#### 4.3.3. Economic Analysis of Slurry

Material cost control is an important part of the actual project management, the current market price of water reducer is 60 US dollars per ton, compared to ordinary cement (180 US dollars per ton), so cost analysis is particularly important. Table 7 calculates the cost price per ton of slurry for the two recommended mix ratios. According to the data, the cost of ordinary cement grout with a water–cement ratio of 0.5 is US $40.25 per ton, while the cost of cement grout with a water–cement ratio of 0.25 and 1.85% water reducing agent added is US $50.19 per ton. It can be seen that the material cost of the cement grout with the recommended mix ratio in this paper is 24.7% higher than that of the ordinary cement grout recommended by the code.

## 5. Conclusions

(1) Increasing the water–cement ratio and the dosage of the water-reducing agent can enhance the flowability of the cementitious grout. However, when the water–cement ratio is relatively high, the effectiveness of the water-reducing admixture in improving the flowability of the grout can be reduced.
(2) The stone-formation rate of the grout decreases with an increase in the dosage of the water-reducing agent. Additionally, the influence of the water–cement ratio on the stone-formation rate is defined by a critical threshold. When the water–cement ratio exceeds this threshold value, the stone-formation rate of the grout decreases. Furthermore, the critical water–cement ratio threshold decreases as the dosage of the water-reducing agent increases.
(3) Without the addition of a water-reducing agent, as the water–cement ratio increases, the overall integrity of the grout stone decreases, and the porosity of the grout stone surface increases. A high porosity of the grout stone is unfavorable for the bonding of the grout with anchor rods and the surrounding rock. When the water–cement ratio remains constant, the addition of a water-reducing agent changes the surface pores of the grout stone from “evenly distributed small pores” to “localized large pores”.
(4) Using the multi-objective optimization approach in Matlab, the proposed recommended optimal mixture composition for the delayed-setting cementitious grout is a water–cement ratio of 0.25 and water-reducing agent dosage of 1.5%.

## Figures and Tables

**Table 1:** Classification of the flow state of a single cement slurry.

Flow State	Fluidity (mm)	Morphology	Draft
Plasticity	60–70	Upright state	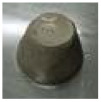
Rheoplasticity	70–120	Conical	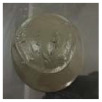
Fluid	≥120	Cake-shaped	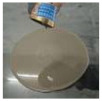

**Table 2:** Physical properties of the P.O42.5 cement.

Physical Property	Strength
3-day bending strength	4.2 MPa
28-day bending strength	7.4 MPa
3-day compressive strength	23.5 MPa
28-day compressive strength	44.2 s
Initial setting time	225 s
Final setting time	263 s
Stability	1.5

**Table 3:** Performance test variable of the cement paste.

Water-Reducing Agent Content	Water–Cement Ratio
0%	0.25, 0.4, 0.5, 0.6, 0.7
1.0%	
2.0%	

**Table 4:** Limiting water–cement ratio and the maximum stone rate at various water-reducer dosages.

Dosage of Water-Reducing Agent	Threshold of Water–Cement Ratio	Maximum Stone Rate
0%	0.3	97.93%
1.0%	0.3	97.73%
2.0%	0.25	99.1%

**Table 5:** Evaluation of the fitting results and the performance.

Variable	Expression	R^2^	Significance
*f*_Fluidity_	−61.1 + 506.829*X*_1_ + 10,410*X*_2_	0.873	<0.001
*f*_Stone rate_	120.638 − 62.384*X*_1_ − 536.7*X*_2_	0.818	<0.001
*f*_Initial setting time_	35.105 + 890.458*X*_1_ + 29,342.244*X*_2_	0.958	<0.001
*f*_Final setting time_	−10.167 + 1005.366*X*_1_ + 39,450*X*_2_	0.974	<0.001
*f*_28-d compressive strength_	220.367 − 315.203*X*_1_ − 320*X*_2_	0.957	<0.001

**Table 6:** Test verification of the optimal cement slurry ratio.

Group	Fluidity	Stone Rate	Initial Setting Time	Final Setting Time	28-Day Compressive Strength
Theoretical	221.7 mm	96.9%	697 min	833 min	136.77 MPa
Experimental	261 mm	99.24%	827 min	875 min	140 MPa
Error	17.7%	2.41%	18.7%	5.04%	2.36%

**Table 7:** Comparative experimental parameters.

Group	Water–Cement Ratio	Dosage of Water-Reducing Agent	Fluidity	Stone Rate	Initial Setting Time	Final Setting Time	28-Day Compressive Strength	Chloride Ion Content	Price per Ton
This study	0.25	1.5%	261 mm	99.24%	827 min	875 min	140 MPa	0.025%	$50.19
Specification	0.5	0%	173 mm	92%	497 min	527 min	57 MPa	0.009%	$40.25

## Data Availability

Not applicable.

## References

[1] da Silva C.C.C., Bernaud D., Real M.D.V., Maghous S. (2023). Reliability analysis of bolt-supported tunnels regarded as homogenized structures. Int. J. Numer. Anal. Methods Geomech..

[2] Azad M.A., Naithani A.K., Shekhar S., Ahmad S., Singh S.K. (2023). Tunnel Support Validation Using Numerical Modelling: A Case Study from NW, Himalaya, India. Geotech. Geol. Eng..

[3] Majumder D., Viladkar M.N., Singh M. (2022). Numerical Modelling of Tunnels Excavated in Squeezing Ground Condition: A Case Study. Arab. J. Sci. Eng..

[4] Riaz A., Jamil S.M., Asif M., Akhtar K. (2016). Tunnel support design by comparison of empirical and finite element analysis of the Nahakki tunnel in mohmand agency, pakistan. Stud. Geotech. Mech..

[5] Li P., Chen Y., Huang J., Wang X., Liu J., Wu J. (2023). Design principles of prestressed anchors for tunnels considering bearing arch effect. Comput. Geotech..

[6] China National Railway Group (2020). Technical Specification for Bolt Support of Railway Tunnel.

[7] China Metallurgical Construction Association (2015). Technical Specification for Geotechnical Bolt and Shotcrete Support Engineering.

[8] Skripkiūnas G., Kičaitė A., Justnes H., Pundienė I. (2021). Effect of Calcium Nitrate on the Properties of Portland–Limestone Cement-Based Concrete Cured at Low Temperature. Materials.

[9] Jing H., Xu M., Gao M., Li M., Dai S. (2022). Effect of Compounding Retarder and PCE on the Early Properties and Hydration of High-Belite Sulphoaluminate Cement. Appl. Sci..

[10] Mollamahmutoğlu M., Avci E. (2015). Ultrafine Portland cement grouting performance with or without additives. KSCE J. Civ. Eng..

[11] Shevchenko V.V., Kotsay G.N. (2021). Influence of Glass Powder Additives on the Hydration Process of Portland Cement. Glass Phys. Chem..

[12] Baltazar L.G., Henriques F.M., Cidade M.T. (2020). Effects of Polypropylene Fibers and Measurement Methods on the Yield Stress of Grouts for the Consolidation of Heritage Masonry Walls. Fluids.

[13] Alrshoudi F., Mohammadhosseini H., Alyousef R., Md. Tahir M., Alabduljabbar H., Mustafa Mohamed A. (2020). The Impact Resistance and Deformation Performance of Novel Pre-Packed Aggregate Concrete Reinforced with Waste Polypropylene Fibres. Crystals.

[14] Meireles P.D., Pereira D.S., Melo M.A., Braga R.M., Freitas J.C., Melo D.M., Silvestre F.R.S. (2019). Technical evaluation of calcium sulphate α-hemihydrate in oilwell application: An alternative to reduce the environmental impacts of Portland cement. J. Clean. Prod..

[15] You C., Qian J., Qin J., Wang H., Wang Q., Ye Z. (2015). Effect of early hydration temperature on hydration product and strength development of magnesium phosphate cement (mpc). Cem. Concr. Res..

[16] Haque M.A., Chen B. (2019). Research progresses on magnesium phosphate cement: A review. Constr. Build. Mater..

[17] Ataie F.F. (2019). Influence of Cementitious System Composition on the Retarding Effects of Borax and Zinc Oxide. Materials.

[18] Murat M., Eyubhan A. (2021). Strength and permeability of boric acid-tempered ultrafine cement grouted sand. Constr. Build. Mater..

[19] Mezhov A., Shir I.B., Schmidt A., Kovler K., Diesendruck C.E. (2022). Retardation mechanism of cement hydration by a comb polyphosphate superplasticizer. Constr. Build. Mater..

[20] Zhu W., Feng Q., Luo Q., Bai X., Chen K., Lin X. (2022). Effect of a specific PCE superplasticizer on the initial dissolution and early hydration of Portland cement. J. Build. Eng..

[21] Padovnik A., Bokan-Bosiljkov V. (2021). The Influence of Dry Hydrated Limes on the Fresh and Hardened Properties of Architectural Injection Grout. Materials.

[22] Vasumithran M., Anand K., Sathyan D. (2020). Effects of fillers on the properties of cement grouts. Constr. Build. Mater..

[23] Lin R., Yang L., Li S., Li R., Sheng X., Song G. (2020). Influences of polymers on the properties of cement-sodium silicate grouts with a high water-binder ratio. J. Ceram. Process. Res..

[24] Qin T., Ni Y., Chen W., Tao L. (2023). Optimization of Composite Grouting Material Proportioning Based on Regression Analysis Method. Sustainability.

[25] Aghaee K., Sposito R., Thienel K.C., Khayat K.H. (2023). Effect of additional water or superplasticizer on key characteristics of cement paste made with superabsorbent polymer and other shrinkage mitigating materials. Cem. Concr. Compos..

